# The impact of nuts consumption on glucose/insulin homeostasis and inflammation markers mediated by adiposity factors among American adults

**DOI:** 10.18632/oncotarget.25168

**Published:** 2018-07-27

**Authors:** Mohsen Mazidi, Hassan Vatanparast, Niki Katsiki, Maciej Banach

**Affiliations:** ^1^ Key State Laboratory of Molecular Developmental Biology, Institute of Genetics and Developmental Biology, Chinese Academy of Sciences, Chaoyang, China; ^2^ Institute of Genetics and Developmental Biology, International College, University of Chinese Academy of Science (IC-UCAS), Chaoyang, China; ^3^ Health Sciences E-Wing, Saskatoon SK, Canada; ^4^ Second Propedeutic Department of Internal Medicine, Medical School, Aristotle University of Thessaloniki, Hippokration Hospital, Thessaloniki, Greece; ^5^ Department of Hypertension, Chair of Nephrology and Hypertension, Medical University of Lodz, Lodz, Poland; ^6^ Polish Mother's Memorial Hospital Research Institute (PMMHRI), Lodz, Poland; ^7^ Cardiovascular Research Centre, University of Zielona Gora, Zielona Gora, Poland

**Keywords:** nut, insulin homeostasis, glucose homeostasis, inflammation, mediation analysis

## Abstract

**Background:**

Inconclusive results have been published regarding the impact of nut consumption on glucose/insulin homeostasis and inflammatory factors. Furthermore, it remains unanswered whether adiposity factors could mediate the association between nut consumption, glucose/insulin homeostasis and inflammatory markers; this is what the current study aims to investigate.

**Results:**

From a total of 16,784 individuals, 48.2% participants were men; overall mean age was 47.2 years. Age-, sex-, energy intake and race-adjusted mean of serum C-reactive protein (CRP)(0.49 to 0.26 mg/dl), apolipoprotein-β (apo- β) (95.6 to 90.8 mg/dl), glucose/insulin homeostasis parameters and triglyceride-glucose index (TyG) index (8.32 to 7.95) significantly decreased as the quartile of nut intake increased (all *p* < 0.001). We found that all evaluated potential mediators had significant and positive associations with markers of glucose/insulin homeostasis or inflammation (all *p* < 0.001). With regard to BMI, the mediated effects were significant for the associations between nut consumption and CRP, fasting blood glucose, insulin, hemoglobin A1c (HbA1c), triglyceride to high-density lipoprotein (TG:HDL) ratio and TyG index (all *p* < 0.001). As for WC, it had mediator impact on CRP, fasting blood glucose, HbA1c, TG:HDL ratio and TyG index (all *p* < 0.001). apVAT played no mediation role for any association (all *p* > 0.05).

**Conclusions:**

This is the first study which quantify the role of nut consumption on inflammatory and glucose/insulin homeostasis markers. Nut intake was inversely associated with inflammatory and glucose/insulin homeostasis markers. Certain adiposity indexes (i.e. BMI and WC) mediated these associations. These findings convey an important message for the crucial role of weight management with dietary recommendations.

**Method:**

We extracted data from the National Health and Nutrition Examination Survey (2005–2010) on nut consumption to evaluate the association between nut intake and markers of glucose/insulin homeostasis and inflammation. We assessed whether this link, if any, is mediated or affected by adiposity factors, including body mass index (BMI), waist circumference (WC, marker of central adiposity), anthropometrically predicted visceral adipose tissue (apVAT), visceral adiposity index (VAI, indicator of adipose distribution) and lipid accumulation product (LPA, novel index of central lipid accumulation). Analysis of co-variance and conceptus causal mediation analysis were conducted based on survey design and sample weights.

## INTRODUCTION

Nuts are widely available around the world and contain many bioactive compounds, including fiber, vegetable protein, minerals, phytosterols, and phenolic compounds [1]. Earlier experiments reported that the cardioprotective properties of nuts consumption might be attributed to its impact on insulin sensitivity and anti-inflammatory effects of nuts [2, 3] It has also been suggested that nuts have a favourable impact on cholesterol [1]. Nuts also contain a high proportion of unsaturated fatty acids, and nut consumption has been inversely associated with circulating inflammatory cytokines and positively associated with plasma adiponectin [1–3]. In this context, a long-term observational study found that nuts consumption was related to higher adiponectin levels [4, 5]. However, it's still not clear if this happened because of body weight lowering (as a secondary effect) or as a primary effect. Furthermore, previous studies suggested that nuts consumption is related to improvements in inflammatory markers including C-reactive protein (CRP), an independent risk factor of cardiovascular disease (CVD) [6, 7]. However, there are also conflicting results regarding the effects of nuts on inflammatory markers; for example, one study reported that a high soy isoflavone diet increased plasma level of interleukin (IL)-6 in women [8]. On the other hand, in a meta-analysis of 20 randomized control trials. we have recently reported that nut consumption has no significant effect on CRP, IL-6 and IL-10 [5].

Available research has demonstrated that nuts are associated with beneficial glycemic responses in healthy individuals. For example, almonds reduced the glycemic impact, calculated as the incremental area under the 2-hour blood glucose curve, of carbohydrate foods in a dose-dependent manner [9]. Moreover it has been reported that pistachio nuts can attenuate the relative glycemic response when taken with a carbohydrate meal (co-consumed) [10]; and another study stated that mixed nuts (30, 60 and 90 g) were demonstrated to have a dose-dependent effect on the glycemic response [11]. Additionally to the results of several studies that suggested that nuts may play a protective role in type 2 diabetes, a significant association between nut consumption and the risk of type 2 diabetes was also not found in a meta-analysis, in which the authors included case-control, cohort and clinical trials [12].

Several cross-sectional and prospective epidemiological studies indicated an inverse association between the frequency of nut consumption and obesity [13, 14]. The protective effect of nuts may in part be explained by the favourable impact of nuts on serum lipids [15], oxidative stress [16] and markers of inflammation [17]. Several studies confirmed the link between adiposity and CRP levels [18, 19]. In this context, body mass index (BMI) and waist circumference (WC) were significantly associated with CRP [20]. Obesity was also associated with higher CRP levels in women with rheumatoid arthritis [21]. It is well-known that an association prevails between obesity and diabetes [22]. Furthermore, it has been stated that obesity plays a key role in the pathophysiology of type 2 diabetes and that early treatment of obesity is essential in the prevention and treatment of type 2 diabetes [22]. A national survey indicated that increased WC might be independently related to the risk of diabetes [23].

It is difficult to evaluate and unravel mechanisms explaining the links between nut intake, inflammation, and glucose control using a simple regression analysis [24]. From a statistical standpoint, mediation analysis can be used to explore and quantify the extent to which the relationship between an exposure and an outcome of interest, occurs through the effect of a third variable [24]. The traditional approach to mediation analysis tends to produce a bias when there is uncontrolled mediator-outcome confounding or an interaction between exposure and mediator. With the use of the counterfactual framework in causal mediation analysis, unbiased valid estimates of direct and indirect effects can be obtained [24]. In this contest, mediation analysis could clarify the role of adiposity underlying the relationship between nut consumption and glucose/insulin homeostasis or inflammatory markers [25]. Furthermore, the degree of mediation may be different between different adiposity indexes [26]. For example, in a prospective cohort study (Health Professionals Follow-Up Study) of 27,270 men evaluating the role of BMI, WC, and waist-to-hip ratio (WHR) in predicting type 2 diabetes [26], they reported that both abdominal and overall adiposity were good predictors, but WC was a better predictor than WHR [26]. Moreover, we have recently investigated the association of triglycerides/glucose index (TyG index), apVAT, lipid accumulation product (LAP), visceral adiposity index (VAI) and triglycerides (TG):high density lipoprotein - cholesterol (HDL-C) ratio with insulin resistance (IR) in adult Americans [27], reporting that, among these markers, apVAT had the highest specificity as a marker of IR [27].

In general, it is unclear to what extent nut consumption may affect cardiometabolic risk profile and particular glucose/insulin homeostasis and inflammation. We hypothesized that a higher nut consumption (including peanuts, tree nuts, and seeds) would be associated with favourable concentrations of inflammatory and glucose/insulin homeostasis biomarkers among adults and that these associations would be in part or fully mediated by adiposity factors. Due to a confusing evidence, we aimed to evaluate the association between nut consumption and glucose/ insulin homeostasis and inflammation parameters, and in the same time assess the mediation effect of different adiposity indexes on the observed associations by applying “*causal mediation analysis*”.

## RESULTS

### General characteristics

A total of 16,784 individuals met the criteria for inclusion in the current analyses. Their characteristics are summarised in Table 1. Overall, 48.2% participants were men and 51.8% were women; their mean age was 47.2 years. Non-Hispanic white (69.4%) was the largest racial group and other Hispanic (4.5%) the smallest racial group. Furthermore, 56.1% of the participants were married.

**Table 1:** Demographic and clinical characteristics of the participants

Characteristics		Overall	*P*-value
**Sex**	**Men (%)**	48.2%	<0.001
	**Women (%)**	51.8%	
**Age (Years)**		47.2 ± 1.3	
**Race/Ethnicity**	**White (non-Hispanic) (%)**	69.3%	<0.001
	**Non-Hispanic Black (%)**	11.9%	
	**Mexican-American (%)**	8.0%	
	**Other Hispanic (%)**	4.4%	
	**Other (%)**	6.3%	
**Marital Status**	**Married (%)**	55.9%	<0.001
	**Widowed (%)**	16.9%	
	**Divorced (%)**	10.9%	
	**Never married (%)**	17.8%	
**Body mass index (kg/m^2^)**		28.1 ± 0.02	
**Waist circumference (cm)**		97.2 ± 0.29	
**Anthropometrically Predicted Visceral Adipose Tissue**		165.9 ± 2.36	
**TyG index**		7.92 ± 0.01	
**TG:HDL ratio**		3.5 ± 0.03	
**Serum CRP (mg/dL)**		0.49 ± 0.02	
**Serum apolipoprotein B (mg/dL)**		34.9 ± 0.82	
**Fasting blood glucose (mg/dL)**		101.4 ± 1.26	
**Plasma insulin (uU/mL)**		2.34 ± 0.02	
**HOMA-IR**		0.86 ± 0.03	
**HOMA-B**		4.72 ± 0.04	
**HbA1c (%)**		5.42 ± 0.09	
**2 h Blood Glucose (mg/dL)**		119.3 ± 1.26	
**Visceral Adiposity Index**		2.4 ± 0.001	
**Lipid accumulation product**		66.3 ± 0.93	

Abbreviations: HOMA-IR: Homeostatic model assessment of insulin resistance; HOMA-B: Homeostatic model assessment of β-cell function, HbA1c: glycated haemoglobin A1c, TyG index: triglyceride-glucose index, HDL: high-density lipoprotein, CRP:C-reactive protein.

Values are expressed as estimated mean and standard error or percent.

We applied ANCOVA to calculate the age-, sex-, energy intake and race-adjusted mean of markers of insulin resistance and inflammation across quartiles of nut consumption. Serum CRP, apolipoprotein B (apo B), fasting glucose, insulin, HOMA-IR, HOMA-B, HbA1c (%), 2 h blood glucose and TyG index significantly decreased as the quartile of nut intake increased (all *p* < 0.001, Table 2).

**Table 2:** Age-, gender-, energy intake, and race-adjusted mean of markers of insulin resistance and inflammation across quartiles of nut consumption

Variables	Quarters of nut consumption				*p* –value^a^
	1	2	3	4	
*n*	4196	4180	4201	4297	
**Serum CRP (mg/dL)**	0.49 ± 0.01	0.42 ± 0.02	0.35 ± 0.02	0.26 ± 0.01	<0.001
**Serum apolipoprotein B (mg/dL)**	95.6 ± 0.92	94.1 ± 1.01	93.9 ± 0.82	90.8 ± 0.93	<0.001
**Fasting blood glucose (mg/dL)**	101.3 ± 0.82	99.6 ± 0.62	98.8 ± 0.34	96.3 ± 0.12	<0.001
**Plasma insulin (uU/mL)**	2.36 ± 0.01	2.28 ± 0.02	2.19 ± 0.03	2.00 ± 0.04	<0.001
**HOMA-IR**	1.16 ± 0.02	1.12 ± 0.01	1.00 ± 0.01	0.82 ± 0.05	<0.001
**HOMA-B**	5.01 ± 0.03	4.93 ± 0.02	4.71 ± 0.01	4.33 ± 0.03	<0.001
**HbA1c (%)**	5.94 ± 0.02	5.83 ± 0.01	5.52 ± 0.01	5.26 ± 0.02	<0.001
**2 h blood glucose (mg/dL)**	132.9 ± 1.26	128.6 ± 2.32	112.8 ± 1.92	102.9 ± 1.08	<0.001
**TyG index**	8.32 ± 0.01	8.12 ± 0.02	8.02 ± 0.02	7.95 ± 0.03	<0.001
**TG:HDL**	3.4 ± 0.01	3.3 ± 0.02	3.2 ± 0.05	2.9 ± 0.03	<0.001

Abbreviations: HOMA-IR: Homeostatic model assessment of insulin resistance; HOMA-B: Homeostatic model assessment of β-cell function, HbA1c: glycated haemoglobin A1c, TyG index: triglyceride-glucose index, HDL: high-density lipoprotein, CRP: C-reactive protein.

All estimates were adjusted for age, sex, race/ethnicity, educational, smoking and level of physical activity. Regression coefficients α, β, γ and γ’ are shown in Supplementary Figure 1.

Values are expressed as estimated mean and standard error.

^a^*p*-values for linear trend across quartiles of CRP; variables were compared across quartiles of CRP using analysis of covariance (ANCOVA).

### Association between nut consumption, BMI, WC, apVAT, LAP, VAI and markers of glucose/insulin homeostasis and inflammation

In the “action theory” (Supplementary Figure 1), there were significant associations between BMI, WC, apVAT, LAP and VAI with nut consumption (BMI = β: –0.110, *p* < 0.001, WC = β: –0.225, *p* < 0.001, apVAT = β: –0.368, *p* = 0.658, LA*P* = β: –0.012, *p* < 0.001, VAI = β: –0.015, *p* < 0.001, Table 3).

**Table 3:** Estimates of regression coefficients (95% CIs) for the association between nut consumption, BMI, WC, apVAT, LAP and VAI (action theory), and markers of insulin resistance and inflammation (total effect) among US adults in the NHANES

Mediator	Estimate	95% CI	*P*
***BMI***	–0.110	–0.171 to –0.04	<0.001
**WC**	–0.225	–0.40to –0.10	<0.001
**apVAT**	–0.368	–1.39 to 0.38	0.658
**VAI**	–0.015	–0.023 to –0.07	<0.001
**LAP**	–0.012	–0.021 to 0.003	<0.001
***Outcome***			
**Serum CRP (mg/dL)**	–0.040	–0.53 to –0.28	<0.001
**Fasting blood glucose (mg/dL)**	–0.509	–0.86 to –0.15	<0.001
**Plasma insulin (uU/mL)**	–0.018	–0.028 to –0.005	<0.001
**HOMA-IR**	–0.021	–0.03 to –0.009	<0.001
**HOMA-B**	–0.009	–0.020 to 0.02	0.235
**HbA1c (%)**	–0.019	–0.028 to –0.09	<0.001
**2 h blood glucose (mg/dL)**	–0.964	–1.73 to –0.19	<0.001
**TyG index**	–0.010	–0.017 to –0.003	<0.001
**Serum apolipoprotein B (mg/dL)**	–0.006	–0.375 to 0.369	0.325
**TG:HDL ratio**	–0.039	–0.085 to 0.007	0.094

Abbreviations: CI: confidence interval, BMI: body mass index, WC: waist circumference, apVAT: anthropometrically-predicted visceral adipose tissue, HOMA-IR: Homeostatic model assessment of insulin resistance; HOMA-B: Homeostatic model assessment of β-cell function, HbA1c: glycated haemoglobin A1c, TyG index: triglyceride-glucose index, HDL: high-density lipoprotein, CRP: C-reactive protein.

All estimates were adjusted for age, sex, race/ethnicity, educational, smoking and level of physical activity. Regression coefficients α, β, γ and γ’ are shown in Supplementary Figure 1.

Furthermore, we also calculated the “total effect” (Supplementary Figure 1) by examining the association between nut consumption and markers of glucose/insulin homeostasis or inflammation in multivariable models without adjusting for potential mediators. It was shown that except for HOMA-B (*p* = 0.235), serum apo B (*p* = 0.325) and TG:HDL ratio (*p* = 0.094), the rest of the markers of glucose/insulin homeostasis and inflammation were negatively and significantly associated with nut consumption. Furthermore, 2 h blood glucose (β: –0.964) had the strongest association, followed by fasting blood glucose and CRP, while TyG index (β: –0.010) had the weakest association with nut consumption (all *p* < 0.001, Table 3).

In the “conceptual theory” (Supplementary Figure 1), referring to the links between mediators (BMI, WC, apVAT, LAP and VAI) and markers of glucose/insulin homeostasis or inflammation, we found that all evaluated potential mediators had significant and positive associations with markers of glucose/insulin homeostasis or inflammation (all *p* < 0.001, Table 4).

**Table 4:** Estimates of regression coefficients (95% CIs) for the association between BMI, WC, apVAT and VAI with markers of insulin resistance and inflammation (conceptual theory) among US adults in the NHANES

Outcomes	BMI		WC		apVAT		VAI		LAP	
	Estimate	95% CI	Estimate	95% CI	Estimate	95% CI	Estimate	95% CI	Estimate	95% CI
Serum CRP (mg/dL)	0.082	0.080–0.085	0.037	0.036–0.038	0.0093	0.0089–0.0096	0.40	0.38–0.42	0.56	0.52–0.56
Serum apolipoprotein B (mg/dL)	0.54	0.46–0.63	0.29	0.26–0.33	0.091	0.073–0.102	14.23	13.95–15.26	13.62	12.52–4.63
Fasting blood glucose (mg/dl)	0.77	0.69–0.88	0.36	0.32–0.40	0.079	0.064–0.095	8.25	7.62–9.12	7.42	6.39–8.54
Plasma Insulin (μU/mL)	0.056	0.054–0.059	0.025	0.024–0.026	0.006	0.005–0.007	0.40	0.38–0.42	0.44	0.43–0.46
HOMA-IR	0.063	0.061–0.065	0.029	0.028–0.030	0.007	0.006–0.008	0.47	0.44–0.49	0.51	0.49–0.52
HOMA-B	0.036	0.034–0.039	0.017	0.016–0.018	0.004	0.003–0.005	0.23	0.21–0.25	0.28	0.26–0.30
HbA1c (%)	0.025	0.023–0.027	0.011	0.010–0.012	0.002	0.001–0.003	0.21	0.19–0.23	0.20	0.19–0.22
2-h blood glucose (mg/dL)	1.42	1.23–1.61	0.67	0.59–0.75	0.19	0.16–0.23	17.45	15.62–18.32	15.7	14.2–17.3
TyG index	0.029	0.027–0.031	0.014	0.013–0.015	0.004	0.003–0.005	0.77	0.72–0.80	0.64	0.63–0.66
TG:HDL ratio	0.111	0.101–0.122	0.057	0.052–0.061	0.014	0.013–0.016	4.22	4.16–4.29	3.10	3.05–3.16

Abbreviations: CI: confidence interval, BMI: body mass index, WC: waist circumference, apVAT: anthropometrically-predicted visceral adipose tissue, HOMA-IR: Homeostatic model assessment of insulin resistance; HOMA-B: Homeostatic model assessment of β-cell function, HbA1c: glycated haemoglobin A1c, TyG index: triglyceride-glucose index, HDL: high-density lipoprotein, CRP: C-reactive protein.

All estimates were adjusted for age, sex, race/ethnicity, educational, smoking and level of physical activity. Regression coefficients α, β, γ and γ’ are shown in Supplementary Figure 1.

### Direct and indirect effects of nut consumption on markers of insulin resistance and inflammation with BMI, WC and apVAT as mediators

Table 5 shows the direct effect, indirect effect, proportion of mediation effect, and Sobel statistics for testing indirect effects of nut consumption on markers of insulin resistance and inflammation with several adiposity markers as mediators. With regard to BMI, the mediated effects were significant for the associations between nut consumption and CRP, fasting blood glucose, plasma insulin, HbA1c, TG:HDL ratio and TyG index (all *p* < 0.001). As for WC, it had mediator impact on CRP, fasting blood glucose, HbA1c, TG:HDL ratio and TyG index (all *p* < 0.001). Out of our expectation, apVAT played no mediation role for any association (all p > 0.05). In contrast, VAI played a mediator role for all of the variables except for serum apo B (*p* = 0.415), as did LAP with the exception of the TyG index (*p* = 0.241).

**Table 5:** Direct and indirect effects of nut consumption on markers of insulin resistance and inflammation with BMI, WC, apVAT, VAI and LAP as mediators among US adults

Mediator and outcomes	Direct effect (γ')		Indirect effect (α#β)		Proportion of mediation, %
	Estimate	*P*	Estimate	Sobel test statistic	
**BMI**					
Serum CRP (mg/dl)	−0.032^*^	<0.001	−0.008^*^	<0.001	20.1%
Serum apolipoprotein B (mg/dL)	−0.005	0.977	−0.024	0.352	81.1%
Fasting blood glucose (mg/dl)	−0.402^*^	<0.001	−0.091^*^	<0.001	18.4%
Plasma Insulin (uU/mL)	−0.014^*^	<0.001	−0.003^*^	<0.001	18.0%
HOMA-IR	−0.017^*^	<0.001	−0.003	0.226	17.4%
HOMA-B	−0.006	0.198	−0.002	0.123	22.4%
HbA1c (%)	−0.015^*^	<0.001	−0.002^*^	<0.001	15.6%
2 h Blood glucose (mg/dL)	−0.802^*^	<0.001	−0.094	0.256	10.1%
TyG index	−0.007^*^	<0.001	−0.003^*^	<0.001	29.6%
TG:HDL ratio	−0.027	0.235	−0.014^*^	<0.001	29.3%
**WC**					
Serum CRP (mg/dl)	−0.035^*^	<0.001	−0.008^*^	<0.001	21.6%
Serum apolipoprotein B (mg/dL)	−0.025	0.925	−0.352	0.625	65.2%
Fasting blood glucose (mg/dL)	−0.395^*^	<0.001	−0.100^*^	<0.001	20.6%
Plasma Insulin (uU/mL)	−0.014^*^	<0.001	−0.004	0.352	21.6%
HOMA-IR	−0.017^*^	<0.001	−0.004	0.296	21.2%
HOMA-B	−0.006	0.263	−0.003	0.635	20.3%
HbA1c (%)	−0.015^*^	<0.001	−0.003^*^	<0.001	16.5%
2 h Blood glucose (mg/dL)	−0.832^*^	<0.001	−0.128	0.133	13.4%
TyG index	−0.006^*^	<0.001	−0.003^*^	<0.001	21.6%
TG:HDL ratio	−0.253	0.133	−0.010^*^	<0.001	39.5%
apVAI					
Serum CRP (mg/dL)	−0.231^*^	<0.001	−0.003	0.132	13.6%
Serum apolipoprotein B (mg/dL)	0.562	0.102	−0.103	0.826	12.0%
Fasting blood glucose (mg/dL)	−0.352	0.235	−0.032	0.621	10.1%
Plasma Insulin (uU/mL)	−0.014	0.235	−0.001	0.362	1.25%
HOMA-IR	−0.018^*^	<0.001	−0.001	0.425	10.3%
HOMA-B	−0.001	0.523	−0.0006	0.426	35.6%
HbA1c (%)	−0.006	0.426	0.001	0.235	21.1%
2 h Blood glucose (mg/dL)	−0.900	0.125	−0.138	0.425	13.3%
TyG index	−0.003	0.142	−0.001	0.429	9.2%
TG:HDL ratio	−0.038	0.415	−0.006	0.352	14.3%
**VAI**					
Serum CRP (mg/dL)	−0.035^*^	<0.001	−0.005^*^	<0.001	14.5%
Serum apolipoprotein B (mg/dL)	0.273	0.412	−0.235	0.415	12.6%
Fasting blood glucose (mg/dL)	−0.384^*^	<0.001	−0.133^*^	<0.001	24.1%
Plasma Insulin (uU/mL)	−0.0102^*^	<0.001	−0.008^*^	<0.001	46.5%
HOMA-IR	−0.012^*^	<0.001	−0.010^*^	<0.001	43.6%
HOMA-B	−0.004	0.235	−0.005^*^	<0.001	56.2%
HbA1c (%)	−0.015^*^	<0.001	−0.003^*^	<0.001	17.2%
2 h Blood glucose (mg/dL)	−0.532	0.415	−0.462^*^	<0.001	44.3%
TyG index	0.001	0.362	−0.011^*^	<0.001	13.2%
TG:HDL ratio	0.026	0.096	−0.061^*^	<0.001	75.2%
**LAP**					
Serum CRP (mg/dL)	−0.034^*^	<0.001	−0.006^*^	<0.001	17.1%
Serum apolipoprotein B (mg/dL)	0.161	0.235	−0.221^*^	<0.001	7.6%
Fasting blood glucose (mg/dL)	−0.402^*^	<0.001	−0.090^*^	<0.001	21.3%
Plasma Insulin (uU/mL)	−0.010^*^	<0.001	−0.007^*^	<0.001	37.2%
HOMA-IR	−0.014^*^	<0.001	−0.008^*^	<0.001	36.6%
HOMA-B	−0.004	0.352	−0.003^*^	<0.001	21.2%
HbA1c (%)	−0.015^*^	<0.001	−0.002^*^	<0.001	14.4%
2 h Blood glucose (mg/dL)	−0.632	0.241	−0.365^*^	<0.001	34.2%
TyG index	−0.002	0.425	−0.007	0.241	76.2%
TG:HDL ratio	0.004	0.965	−0.035^*^	<0.001	10.2%

Abbreviations: BMI: body mass index, WC: waist circumference, apVAT: anthropometrically-predicted visceral adipose tissue, HOMA-IR: Homeostatic model assessment of insulin resistance; HOMA-B: Homeostatic model assessment of β-cell function, HbA1c: glycated haemoglobin A1c, TyG index: triglyceride-glucose index, HDL: high-density lipoprotein, CRP: C-reactive protein.

All estimates were adjusted for age, sex, race/ethnicity, educational, smoking and level of physical activity. Regression coefficients α, β, γ and γ’ are shown in Supplementary Figure 1.

Results of the direct effects estimates showed that nut consumption may be associated with CRP, fasting blood glucose, plasma insulin, HOMA-IR, TyG index, HbA1c and 2 h blood glucose levels, even after adjustment for BMI or WC (all *p* < 0.001). CRP and HOMA-IR were associated with nut consumption, even after correction for apVAT (both *p* < 0.001). The same trend was observed for both VAI and LAP, showing that CRP, fasting blood glucose, plasma insulin, HOMA-IR and HbA1c were significantly associated with nut consumption, even after correction for either VAI or LAP adjustments (all *p* < 0.001).

## DISCUSSION

To our knowledge, this is the first study on a nationally representative population with a large sample size that evaluated the mediator role of the several adiposity factors on the association between nut consumption with inflammatory and glucose/insulin homeostasis markers. We found that participants with a higher nut intake had a more cardioprotective profile of glucose/insulin homeostasis and inflammatory markers. Furthermore, adiposity could play a mediator role (with a varied extent, from partial to fully) on the association between nut consumption, inflammatory and glucose/insulin homeostasis parameters.

Our findings suggest that a higher nut consumption may lead to lower CRP levels. In line with our findings, some cross-sectional studies revealed lower circulating concentrations of pro-inflammatory cytokines or endothelial cell adhesion molecules in individuals consuming nuts. For example, a-linolenic acid (18:3(n-3)), extracted from a specific nut was inversely associated with CRP, IL-6, soluble tumor necrosis factor (TNF) receptors 1 and 2 and fibrinogen levels in healthy individuals and/or patients with stable coronary artery disease [45, 46]. The authors reported that a-linolenic acid was extracted from a plant base but they did not mention about the specific sourse of a-linolenic acid. Moreover, individuals with a higher nut consumption presented lower levels of Vascular cell adhesion protein 1 (VCAM-1), Intercellular Adhesion Molecule 1 (ICAM-1)-1, IL-6 and CRP [47]. Previous studies reported the antioxidant and anti-inflammatory properties of nuts [48, 49]. These beneficial effects are attributed to the configuration of nuts (pistachio), which is recognized by a greater level of mono-unsaturated fatty acids (MUFAs), lesser saturated fatty acids, no cholesterol and a suitable amount of proteins, fiber, phytosterols, antioxidants and numerous minerals and vitamins [1, 17]. A growing number of studies investigated the impact of mentioned nutrients on inflammation and oxidative stress; [50–54]. In case of the cardio- protective impact of the nuts, it has been reported that among persons at high cardiovascular risk, a Mediterranean diet supplemented with extra-virgin olive oil or nuts reduced the incidence of major cardiovascular events [55].

Nuts also have PUFA and phytoestrogens (not tree nuts), which are independently related with lower concentrations of inflammatory markers [56]. Furthermore, fiber and glycemic index (GI) are inversely related and it has been reported that low-GI diets may decrease CRP levels [57]. Another long-term observational study found that nuts consumption was related to higher adiponectin levels [4] and a previous cross-sectional study involving 8,105 adults reported that dietary saturated fat decreased across CRP quarters [59]. However, the information on the relation between inflammation and nut consumption is not conclusive. For example, we recently showed in a systematic review and meta-analysis of 20 randomized controlled clinical trials that nut consumption had no significant effect on CRP, IL-6, adiponectin, IL-10, and TNF-α [4, 5]. This discrepancy may be attributed to differences in studied populations, comorbidities at baseline, various ethnicities and different doses of nuts.

The present study also found that individuals with a higher load of nuts in their diet have a more cardioprotective profile of glucose/insulin homeostasis. It has been reported that, in addition to fat and protein, nuts are rich sources of phytates and phenolics, both of which can reduce amylolytic digestion *in vitro* and postprandial glycaemia *in vivo* [58, 59]. In line with our results, some studies found a significant decrease in fasting insulin levels [60, 61] or improvement in insulin resistance [60] following nut consumption. Results from the Nurses’ Health Study cohort [14] but not the Iowa Women's Health Study [62] indicated that consumption of ≥ 5 servings per week of nuts compared with rarely or no intake reduced the risk of developing diabetes (relative risk = 0.73, 95% confidence interval, 0.60–0.89). With regard to the impact of fat on glycaemia, it has been reported that lower total and saturated fat intake was associated with a lower HbA1c [63]. Furthermore, there are studies reporting a higher risk of diabetes for subjects with a higher intake of total and saturated fatty acids [64, 65]. With regard to the role of protein intake on glycaemia, it has been reported that dietary protein does not alter postprandial glycaemia [66]. However, it stimulates a significant postprandial insulin response, which is required for amino acid uptake [67]. In order to maintain euglycaemia, protein concurrently stimulates glucagon secretion, thereby promoting hepatic glucose release and regulation of blood glucose [68].

Nuts also contain approximately 3.3 g of insoluble dietary fiber per ounce [69]. A high intake of dietary fiber (of the more soluble type), higher than the level recommended by the American Diabetes Association, was shown to improve glycaemic control and decrease hyperinsulinaemia as well as plasma lipid concentrations in patients with type 2 diabetes [70]. Other studies reported that long-term nut intake significantly improved glycaemia by reducing fasting insulin levels [60, 61]. Furthermore, the study by Casas-Agustench also showed significant improvements in insulin resistance [60] following nut consumption. Of note, the effects of fibers on glycaemia is unlikely to be the main reason, but this may play a role in cell wall integrity and the digestibility of almond [71]. Low-GI diets can decrease HbA1c [72]. However, there are also conflicting results; for example, Lovejoy *et al.* reported no significant decreases in HbA1c, fasting and 2 h glucose and insulin responses after the consumption of 100 g/day of nuts for 4 weeks [73]. Nevertheless, the study was of relatively short duration to reveal any changes in glycosylated proteins and body weight was slightly increased during the study period [73].

It should be noted that no study have reported a significant reduction in glycated proteins as a marker of long-term glycaemic control [60, 73, 74]. In a randomised cross-over study of 61 healthy subjects that consumed peanuts vs. nut free diet for 12 weeks, despite a greater energy intake during the peanut phase, there were no differences in body composition, possibly due to incomplete nutrient absorption and energy utilization [75]. Some of the inconsistencies in the findings between these trials may be explained by the variations in the health status of the study participants, the sample size and duration of trials, outcome measures and dose of nuts consumed. The favourable effects of nut intake may in part be attributed to the low carbohydrate content of nuts. Furthermore, gastric emptying is reduced by fat and energy load [76, 77]. Therefore, the increasing fat and energy load with an increasing dose of mixed nuts may explain the observed dose-dependent reduction in glycaemia in response to the intake of meals with nuts plus white bread [78]. It has been reported that the glycaemic response of bread can be lowered by the addition of any type of fat [78]. It is also possible that the addition of nuts can decrease carbohydrate absorption or alter the osmotic load and volume of the stomach, all of which could affect the post-prandial glycaemic response. This may relate to the high unsaturated fat content of nuts and their unique physical structure. It is has been suggested that patients with metabolic disorders such as the metabolic syndrome and diabetes may be less responsive to nuts and perhaps other healthy foods [11].

Our study has some strengths. The large sample size afforded adequate statistical power to conduct the complex analysis with little risk of multiple comparisons effects. The quality of metabolic and anthropometric measurements obtained at the NHANES study visit, including WC measurement as a marker of visceral adiposity and a glucose tolerance test as a measure of insulin sensitivity, allowed for the adjustment of these variables with a high degree of precision, potentially providing insight to the pathway by which nut consumption could influence inflammatory markers and insulin/glucose homeostasis. Moreover, the present study focused on mediated effects of different adiposity markers, including not only BMI and WC (which are indicators of general and abdominal obesity) but also apVAT, VAI and LAP in the associations between nut consumption, markers of glucose/insulin homeostasis and inflammation (assessed by causal mediation analyses). As the present study evaluated a representative and large sample size, our results can be extrapolated to the general population.

We are also aware that our analysis has some limitations. The cross-sectional nature precludes any reliable establishment of the sequence of happening between nut consumption, change in adiposity, glucose/insulin homeostasis and inflammation. However, it is unlikely that glucose/insulin homeostasis or inflammation profile can affect nut consumption, whereas the reciprocal relationship between adiposity, glucose/insulin and inflammation has been largely documented. The mediated effect of WC may be affected by BMI, or vice versa, because of the high correlation between WC and BMI (co-linearity). However, given to complex survey design in the present study, it was not feasible to include two mediators simultaneously in a model to test for the mentioned issue [45]. To address this point, we used other adiposity markers, i.e. apVAT, VAI and LAP. Finally, although BMI and WC are commonly used to estimate obesity, these markers may be inaccurate and can lead to biases in measuring adiposity. Therefore, the association between nut consumption and overall adiposity can be underestimated when only BMI or WC is used as a marker of adiposity. For example, BMI, an indirect measure of adiposity, is traditionally weaker than direct measures of adiposity because it does not take into consideration the age, sex, bone structure, fat distribution or muscle mass [79]. To overcome this issue, we also included in our analyses apVAT, VAI and LAP, which are sensitive to age and sex. Furthermore, VAI and LAP are not only adiposity indexes but also more representative of both adiposity and lipid profile.

The findings of our study lead to important clinical and public health messages. The low glycaemic and anti-inflammatory effects of nuts may provide a reason for the inclusion of nuts in diets aimed at reducing the risk of type 2 diabetes, impaired glucose metabolism and CVD, substituting high GI carbohydrates and thus favourably affecting individual metabolism. Furthermore, it may be beneficial to advise people with any of non-communicable diseases to consume nuts instead of carbohydrates as part of their daily diet to reduce the acute, post-prandial glycaemic impact of the meal. Our findings and recommendations are in line with current guidelines by the American Diabetes Association [80] and the International Diabetes Federation [81] which emphasize on the consumption of a healthy diet to control post-meal hyperglycaemia.

## MATERIALS AND METHODS

### Population characteristics

The National Health and Nutrition Examination Survey (NHANES) is a cross-sectional survey conducted by the US National Center for Health Statistics (NCHS) [28]. NHANES survey cycles between 2005–2010 were included in the current study (aged ≥ 18 years, excluding pregnant and lactating respondents, as well as those with missing information on the variables of interest). Data on demographic information were collected through in-home administered questionnaires, while anthropometrical and biochemistry data were collected by trained personnel using mobile exam centers (MEC). More comprehensive data on these procedures are accessible elsewhere [28, 29]. All methods were carried out in accordance with the relevant guidelines and regulations approved by the US NCHS [29–32]. The NCHS Research Ethics Review Board approved the NHANES protocol and consent was obtained from all participants [28]. All methods were performed followed the Declaration of Helsinki regarding ethical standards for research involving human subjects [28].

Homeostatic model assessment of insulin resistance (HOMA-IR) and β-cell function (HOMA-B) were calculated as follows: HOMA-IR = [fasting glucose (nmol/L) * fasting insulin (mU/mL)/22.5], and HOMA-B = [20 × fasting insulin (μU/ml)]/ [fasting glucose (mmol/l) − 3·5] [33]. Triglyceride-glucose (TyG) index was calculated as ln[fasting triglyceride (mg/dl) × glucose (mg/dl)/2] [34]. The anthropometrically predicted visceral adipose tissue (apVAT) was calculated by sex-specific validated equations as: 6 * WC-4.41 * proximal thigh circumference + 1.19 * age – 213.65 for men, 2.15 * WC – 3.63 * proximal thigh + 1.46 * age + 6:22 * BMI -92.713 for women [35]. Visceral adiposity index (VAI) was calculated using sex-specific formulas: for men [WC/39.68 + (1.88 ×BMI)] × (TGs/1.03) × (1.31/high density lipoprotein (HDL)); for women: [WC/36.58 + (1.89 × BMI)] × (TGs/0.81) × (1.52/HDL), where both TGs and HDL levels are expressed in mmol/L [36]. Lipid accumulation product (LPA) was calculated as [WC–65] × [TG] in men, and [WC–58] × [TG] in women [37]. Smoking status was self-reported by the participants.

### Nuts consumption

Dietary intake was assessed via 24 h recall questionnaire obtained by a trained interviewer during the mobile examination center visit with the use of a computer-assisted dietary interview system with standardized probes, i.e. the US Department of Agriculture Automated Multiple-Pass Method (AMPM) [38, 39].

### Statistical analysis

Data were analysed using SPSS^®^ complex sample module version 22.0 (IBM Corp, Armonk, NY, USA). According to the Centre for Disease Control (CDC) guidelines for analysis of complex NHANES datasets, accounting for the masked variance and using the proposed weighting methodology [40], we used means and standard deviations for continuous measures (analysis of variance) and percentages for categorical variables (chi-square). Age, race and sex-adjusted means of insulin resistance and inflammatory markers across the quarters of the nut consumption were computed, using the analysis of covariance (ANCOVA). All tests were two-sided and *p* < 0.05 was the level of significance. We used <0.001 if the *p*-value was lower than 0.001.

In the present study, we assessed the total, direct and indirect effects of nut consumption on markers of insulin resistance and inflammation with BMI, WC, apVAT, LAP and VAI as a mediator by using the counterfactual framework [41, 42]. In this approach, total effect can be decomposed into direct effect (not mediated by BMI, WC, apVAT, LAP, VAI) and indirect effect (mediated by BMI, WC, apVAT, LAP, VAI). The SPSS Macro developed by Preacher and Hayes [43] was used to evaluate the direct effect and indirect effects of nut intake on markers of insulin resistance and inflammation with BMI, WC, apVAT, LAP, VAI as mediators. A product-of-coefficients test was used as it has the potential to detect significant mediation effects in the absence of a significant intervention effect [41, 42]. Utilizing single mediator models, the SPSS macro was used to calculate all regression coefficients which were adjusted for baseline values. In brief, the macro generates output that includes the following steps. Firstly, the total effect (C coefficient) of the intervention on the outcome variable (e.g. markers of insulin resistance and inflammation) is estimated by regressing. The action theory test is then used to examine the effect of the intervention (nut consumption) on the hypothesized mediators (α coefficient, BMI, WC, apVAT, LAP, VAI). The conceptual theory test examines the association between changes in the hypothesized mediators and in the dependent variables (i.e. markers of insulin resistance or inflammation; β coefficient). The program also estimates the direct (γ’ coefficient) and indirect (α#β product of coefficients) effects. The proportion of the mediation effect was calculated using the following equation [α#β/(α#β + γ)]. Full or complete mediation is present when the total effect (the γ-path) is significant, the direct effect (the γ’-path) is non-significant and the indirect effect (the α#β path) is significant, whereas partly or incomplete mediation is present when the direct effect (the γ’-path) is also significant. Inconsistent mediation is present when neither total nor direct effect is significant and the indirect effect (the α#β path) is significant [44].

## CONCLUSIONS

This is the first study which quantify the role of nut consumption on inflammatory and glucose/insulin homeostasis markers. Nut intake was inversely associated with inflammatory and glucose/insulin homeostasis markers. Certain adiposity indexes (i.e. BMI and WC) mediated these associations. These findings convey an important message for the crucial role of weight management by a healthy diet.

## SUPPLEMENTARY MATERIALS FIGURES AND TABLES


