# Supplementary material for: The impact of nuts consumption on glucose/insulin homeostasis and inflammation markers mediated by adiposity factors among American adults
Source: Oncotarget. 2018 Jul 27;9(58):31173–86. doi: 10.18632/oncotarget.25168 (PMC6101284; doi:10.18632/oncotarget.25168)
Supplement: Supplementary file 1 [file oncotarget-09-31173-s001.pdf]

The impact of nuts consumption on glucose/insulin homeostasis and inflammation markers mediated by adiposity factors among american adults

SUPPLEMENTARY MATERIALS

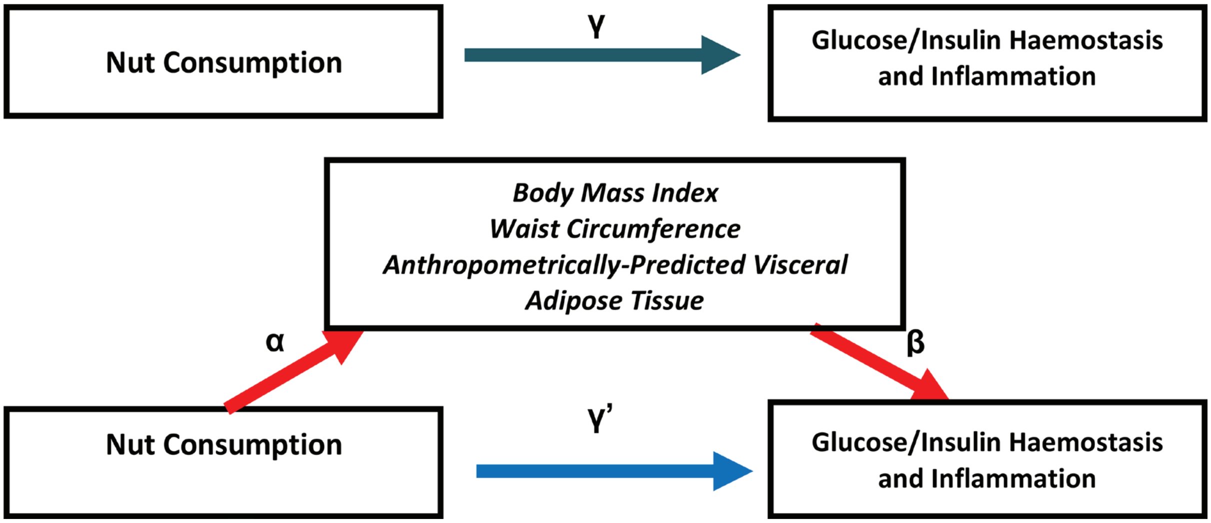

Supplementary Figure 1: Mediation model for the association between nut consumption, glucose/insulin homeostasis and inflammation with body mass index (BMI), waist circumference (WC) and anthropometrically-predicted visceral adipose tissue (apVAT), lipid accumulation product (LAP) and visceral adiposity index (VAI) as mediators. Path α represents the regression coefficient for the association of nut consumption with BMI, WC, apVAT, LAP and VAI. Path β represents the regression coefficient for the association of BMI, WC, apVAT and VAI with glucose/insulin homeostasis and inflammation. The product of regression coefficients α and β represents the mediated effect (indirect effect) of BMI, WC, apVAT, LAP or VAI (α#β). Path γ, represents the direct effect of nut consumption with glucose/insulin homeostasis and inflammation, after adjustment for BMI, WC, apVAT, LAP or VAI. Path γ represents the simple total effect of nut consumption on glucose/insulin homeostasis and inflammation, without adjustment for BMI, WC, apVAT, LAP or VAI.
